# Right Testicular Seminoma With Bilateral Testicular Atrophy in a 44-Year-Old Infertility Patient

**DOI:** 10.7759/cureus.26527

**Published:** 2022-07-03

**Authors:** Delange Augustin, Samuel Junior Orismé, Gina Joachim, Ralph Venderley Pierre, Estimable Luckenson

**Affiliations:** 1 Radiology, Hôpital de l'Université d'Etat d'Haïti, Port-au-Prince, HTI; 2 Urology, Hôpital de l'Université d'Etat d'Haïti, Port-au-Prince, HTI; 3 Département d'Imagerie Medicale, Faculté de Médecine et de Pharmacie, Port-au-Prince, HTI; 4 Radiology, Hôpital de l'Université d'Etat d'Haiti, Port-au-Prince, HTI; 5 Radiodiagnosis, Hôpital de l'Université d'Etat d'Haïti, Port-au-Prince, HTI

**Keywords:** adult male, testicular cancer, infertility, testicular atrophy, testicular seminoma

## Abstract

Testicular cancer is common but curable when diagnosed early. Testicular cancer is often characterized by a painless unilateral testicular mass discovered incidentally. In rare cases, testicular cancer is manifested as testicular atrophy. This case study concerns a 44-year-old patient diagnosed with right testicular seminoma complicated by infertility with bilateral testicular atrophy. In countries where sperm cryopreservation is not feasible for everyone, early detection of testicular atrophy by transscrotal ultrasound could prove effective for rapid intervention to preserve patient fertility in those with asymptomatic intratesticular cancer.

## Introduction

Testicular cancer is the most common malignancy in men aged 15-45 years. With a cure rate of approximately 90% and a five-year survival rate of over 95%, it is one of the most common curable malignancies when identified early and treated appropriately [1]. Testicular cancer comprises 5% of all urological tumors and 1% of all tumors in male patients, and its incidence has doubled over the past 40 years. Its manifestation and management have a long-term impact on patient quality of life [1,2]. Testicular cancer is typically characterized by a painless unilateral testicular mass discovered incidentally. Any solid intratesticular mass should be considered carcinogenic until proven otherwise [1]. In rare cases, testicular cancer manifests as testicular atrophy [3]. We present a rare and challenging case of right testicular seminoma with bilateral testicular atrophy in a 44-year-old man presenting for infertility.

## Case presentation

A 44-year-old man presented for infertility assessment after five years of marriage. He had no medical history apart from a notion of low testicular volume he has had since adolescence. On physical examination, we noted painless and hypotrophic testicles palpated in the bursa, with no evidence of mass or swelling. Our initial diagnosis was bilateral testicular atrophy associated with male infertility of idiopathic etiology.

Semen analysis revealed necrozoospermia and oligozoospermia. Hormone levels showed an increase in follicle-stimulating hormone and luteinizing hormone. Tumor markers revealed increased lactate dehydrogenase (LDH) levels. The patient's beta-human chorionic gonadotropin (B-HCG) and alpha-fetoprotein (AFP) were within reference ranges.

**Figure 1:**
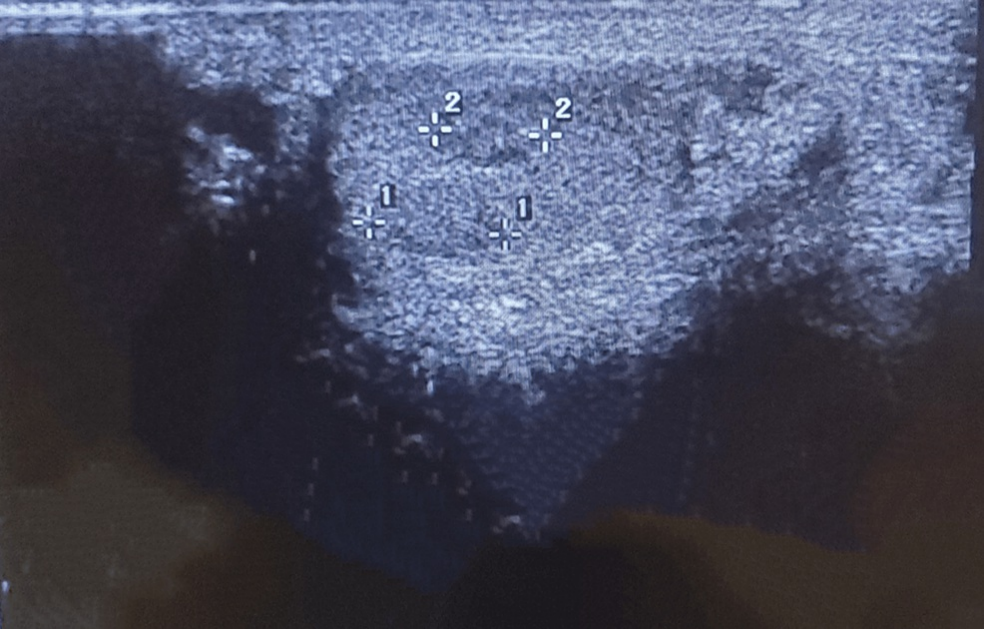
Right intratesticular nodules

**Figure 2:**
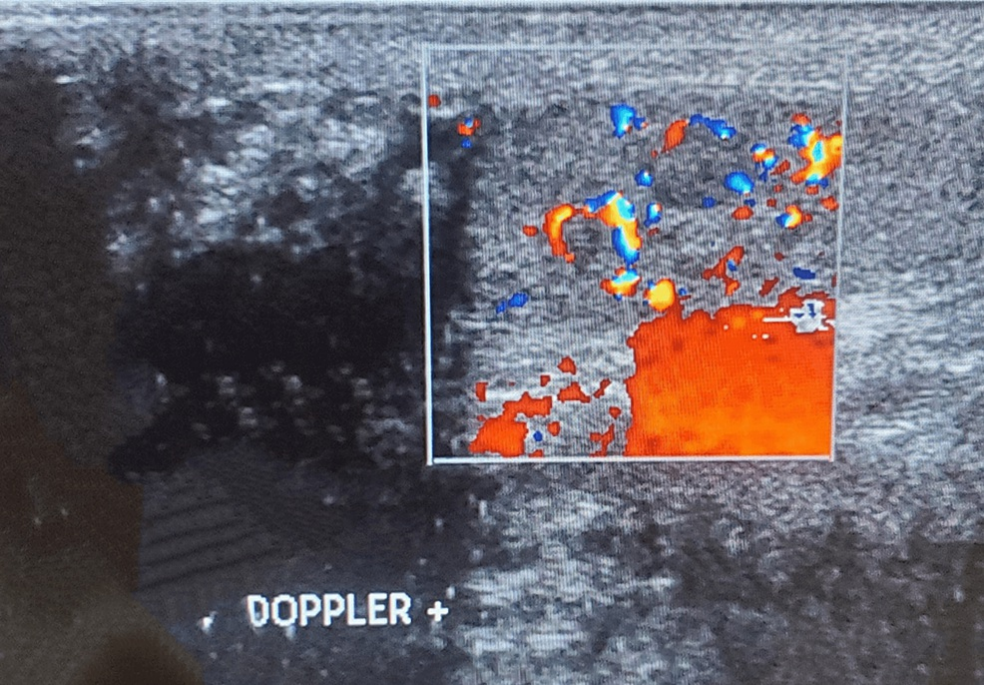
Intratesticular nodules with Doppler

**Figure 3:**
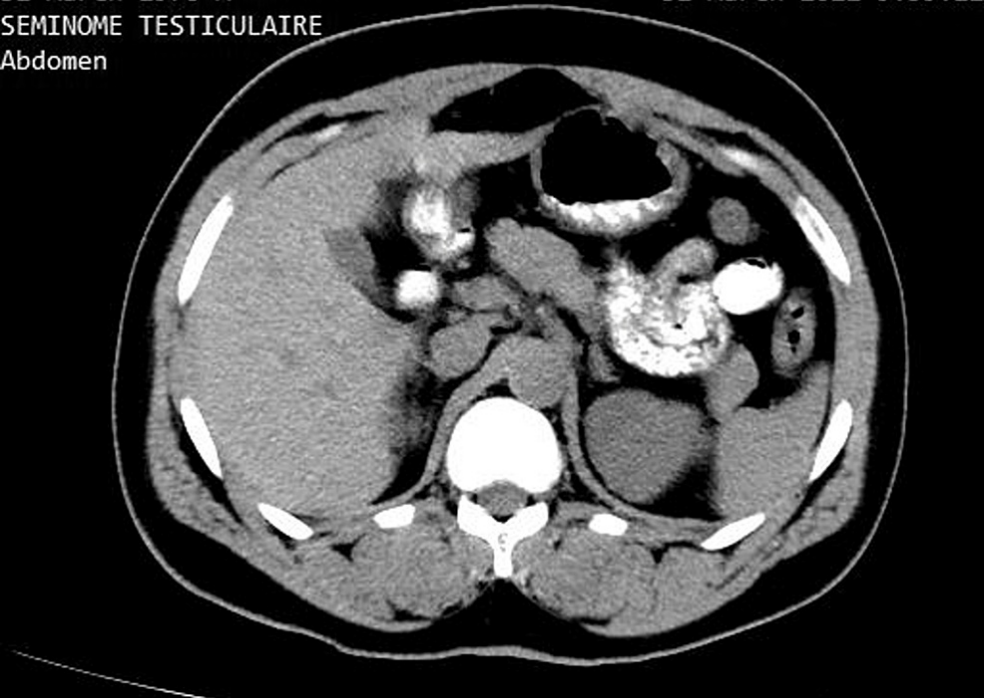
Computed tomography section of the abdomen at the level of the proximal renal arteries showing no metastases to the para-aortic lymph nodes

Inguinoscrotal ultrasound revealed bilateral testicular atrophy with two homogeneous right intratesticular oval nodules. The nodules were hypoechoic, well-circumscribed, and vascularized and measured 7.1 mm and 5.7 mm, respectively (Figures 1, 2). A computed tomography (CT) scan of the abdomen and pelvis revealed no evidence of metastases to the para-aortic lymph nodes at the level of the renal vessels (Figure 3). Therefore, the revised diagnosis was right testicular seminoma stage 1 of the tumor, node, and metastasis (TNM) classification, complicated by infertility with bilateral testicular atrophy. He underwent unilateral right orchiectomy, with continuous follow-up for infertility and ultrasound monitoring of the contralateral testicle every three months.

## Discussion

Testicular cancer most commonly presents as a painless testicular mass or nodule. Upon physical examination, this mass or nodule cannot be separated from the testicle. Patients with atrophic testicles generally experience an increase in the volume of the testicle [4]. Our patient had bilateral testicular atrophy along with two homogeneous right intratesticular nodules revealed by transscrotal ultrasound.

Ultrasound has a sensitivity of 92-98% and a specificity of 95-99.8% for testicular malignancies. Its sensitivity is approximately 100% when combined with physical examination [5]. Seminomas generally appear as a homogeneous intratesticular oval mass that is hypoechoic compared to healthy testicular tissue, which is well-defined, without local invasion, and rarely extends to the paratesticular structures. Internal blood flow is visible on the color Doppler. The chief differential diagnosis is nonseminomatous germ cell tumor, which generally appears heterogeneous and is frequently associated with cystic lesions and calcifications. Our patient's abdominal CT at the level of the proximal renal arteries showed no evidence of metastases to the para-aortic lymph nodes, which are the typical first site of metastases due to the lymphatic drainage of the testes. This also helps rule out testicular lymphoma, the primary differential diagnosis to consider in the presence of para-aortic lymphadenopathy [5].

The possible causes of bilateral testicular atrophy are numerous, and, according to the American Cancer Society, testicular cancers rarely lead to testicular atrophy [3,6]. Testicular cancer can cause testicular atrophy and adversely affect male reproductive health. More than half of men with testicular cancer initially present with oligospermia [7]. The semen analysis in our case revealed zero sperm motility, oligozoospermia, and necrozoospermia.

The diagnosis of seminoma was made according to ultrasound findings and tumor markers; the latter revealed an increase in LDH levels, but B-HCG and AFP were within reference ranges. The markers are essential for the screening and management of testicular cancers. LDH-1 is expressed on chromosome 12p, which is overexpressed in germ cell tumors. Analysis of AFP and B-HCG is seen in many cases of germ cell tumors [8]. Choriocarcinomas frequently express B-HCG, as do approximately 15% of seminomas. AFP is never increased in cases of pure seminoma [9].

The patient's history revealed low testicular volume, observed since puberty. Upon reviewing the literature, germ cell tumors develop following an in utero oncogenic event inducing intratubular germ cell neoplasia [10]. Intratubular germ cell neoplasia comes from a lack of differentiation of gonocytes into spermatogonia [11]. These cells reach an invasive potential after hormonal changes during adolescence. Seminomas consist of transformed germ cells blocked in their differentiation. Embryonal carcinoma cells are similar to undifferentiated stem cells, and their gene expression looks like stem cells and intratubular germ cell neoplasms [12,13].

Seminoma was suspected to be the primary cause of infertility for our patient. An undiagnosed infertility history can also be the cause of the formation of testicular cancer. Walsh et al. reported that men with male factor infertility are three times more likely to develop testicular cancer [14]. Intratubular germ cell neoplasia has been found in 0.4-1.1% of men undergoing testicular biopsy due to infertility [4].

Because his diagnosis was stage 1 seminoma, we managed the patient with unilateral orchiectomy without adjuvant therapy. Although 15-18% of patients with a stage 1 tumor relapse after orchiectomy without adjuvant therapy [15], our patient's risk of relapse was less than 5%, given the absence of a large volume (>4 cm) and no invasion of the rete testis; thus, there was no need for adjuvant therapy. Invasion of both lymphovascular tissue and rete testis carries a 25% risk of relapse, while the chance of relapse is only 4% if neither is involved [16]. Approximately 45-55% of patients with testicular cancer experience azoospermia or oligospermia during treatment or up to two years afterward. Patients wishing to preserve their fertility should be offered sperm cryopreservation before commencing treatment [17]. Unfortunately, the cost of sperm cryopreservation was beyond the patient's means.

While many patients will have sperm recovery after chemotherapy or radiotherapy, predicting which patients will have a return of spermatogenesis is a challenge [7]. Thus, patients with testicular atrophy should benefit from testicular cancer screening using transscrotal ultrasound. Early intervention during puberty or young adulthood would increase the chances of preserving fertility.

## Conclusions

We present a rare right testicular seminoma case complicated by infertility with bilateral testicular atrophy. The patient's condition was treated with unilateral orchiectomy without adjuvant therapy. This case highlights that early detection of testicular atrophy by transscrotal ultrasound could prove effective for rapid intervention to preserve patient fertility in those with asymptomatic intratesticular cancer.
